# Computational Investigation of the Ordered Water System Around Microtubules: Implications for Protein Interactions

**DOI:** 10.3389/fmolb.2022.884043

**Published:** 2022-04-25

**Authors:** Francesco Chierici, Aristide Dogariu, Jack A. Tuszynski

**Affiliations:** ^1^ DIMEAS, Politecnico di Torino, Torino, Italy; ^2^ CREOL, The College of Optics and Photonics, University of Central Florida, Orlando, FL, United States; ^3^ Department of Physics, University of Alberta, Edmonton, AB, Canada

**Keywords:** microtubules, exclusion zone, ordered water layers, Poisson-Boltzmann equation, protein interactions

## Abstract

The existence of an exclusion zone in which particles of a colloidal suspension in water are repelled from hydrophilic surfaces has been experimentally demonstrated in numerous studies, especially in the case of Nafion surfaces. Various explanations have been proposed for the origin of this phenomenon, which is not completely understood yet. In particular, the existence of a fourth phase of water has been proposed by G. Pollack and if this theory is proven correct, its implications on our understanding of the properties of water, especially in biological systems, would be profound and could give rise to new medical therapies. Here, a simple approach based on the linearized Poisson-Boltzmann equation is developed in order to study the repulsive forces mediated by ordered water and involving the following interacting biomolecules: 1) microtubule and a tubulin dimer, 2) two tubulin dimers and 3) a tubulin sheet and a tubulin dimer. The choice of microtubules in this study is motivated because they could be a good candidate for the generation of an exclusion zone in the cell and these models could be a starting point for detailed experimental investigations of this phenomenon.

## Introduction

Recently, the behavior of water at the interface with surfaces has gained a lot of attention (Henderson, 2002). In particular the existence of structural differences between bulk water and water at an interface with hydrophilic surfaces has been proposed by several research groups (Elton et al., 2020). Pollack et al. experimentally demonstrated the presence of a water region near a Nafion surface from which microspheres in colloidal suspension are excluded, which was made clear by observations under the microscope (Zheng and Pollack, 2003). This interface has been termed the exclusion zone (EZ). The EZ water has been shown to extend for as far as hundreds of microns and it has different physical properties with respect to bulk water. In particular, it exhibits a higher refractive index and viscosity and it absorbs electromagnetic energy with a peak at 270 nm (Pollack, 2013). In addition, experiments showed that EZ water is negatively charged, exhibiting a local electrostatic potential of about −200 mV. While it also excludes protons, positive ions can significantly reduce its extension (Zheng and Pollack, 2008) and the interactions generating it seem to have electrostatic origins (Das and Pollack, 2013). Finally, it has been demonstrated that electromagnetic radiation affects the size of the EZ: in particular, IR light is the most effective in expanding it (Chai et al., 2009; Wang and Pollack, 2021). Various explanations have been proposed to provide a physical mechanism for this phenomenon and are reviewed in Elton et al. (2020). Among the others, Pollack advanced the hypothesis of the existence of a fourth phase of water, with properties interpolating between those of ice and liquid water (Pollack, 2013). On the other hand, other research groups proposed EZ water origins from diffusiophoresis (Musa et al., 2013; Schurr et al., 2013). Finally, Van der Waals forces (also called Casimir-Poldor forces) (De Ninno, 2017) and quantum coherence phenomena (Del Giudice et al., 2015) were analyzed as EZ formation theories. Nafion is negatively charged and the most effective way to see the EZ extending from its surface is to use negatively charged suspended particles. Other surfaces, like Polyacrylic acid gel, polyvinyl alcohol gels, metals and biological tissues were tried as well and also neutral and positive particles were placed in colloidal suspensions, but these results were less pronounced (Zheng et al., 2006; Zheng and Pollack, 2008; Elton et al., 2020), supporting the fundamental role of electric interactions, in particular those between negative charges in solution. From experimental results this phenomenon can be easily observed under the microscope at standard laboratory temperature and low ionic concentrations. Increasing the ions concentrations to the mM range reduces the EZ size up to a half (Zheng and Pollack, 2008) and using 2-(N- morpholino)ethanesulfonic acid (MES) at 100 mM to provide polyvalent counterions to the solution does not eliminate the phenomenon but again results are less pronounced (Mercado-Uribe et al., 2021). Also increasing the pH reduces the EZ, that is still detectable at pH ≈ 2 (Mercado-Uribe et al., 2021). Finally, although micrometer-size colloidal particles are usually used, it seems that there is no or very small lower limit to the particle size to be excluded, since the EZ repels also protons (Wang and Pollack, 2021).

If Pollack’s hypothesis is correct, it could have major consequences for biological systems, many of which have hydrophilic surface and virtually all involve interactions with water molecules (Pollack, 2013). Among subcellular structures microtubules (MTs) represent excellent candidates for demonstrating effects of EZ water due to the fact that they form large negatively charged surfaces from which smaller molecules, such as other proteins and ions in the cytoplasm, are excluded. MTs are a key component of the cytoskeleton of eukaryotic cells. They are hollow cylinders with an internal radius of 8.4 nm and external one of 12.5 nm. These polymers are typically made of 13 protofilaments of tubulin dimers and each dimer is made of one α-tubulin and one β-tubulin subunits. Tubulin is a globular protein of molecular mass around 55 kDa, so a tubulin dimer is about 110 kDa and has dimensions 4 × 5 × 8 nm. Moreover, tubulin dimers are highly charged compared to other proteins, with a bare electric charge of about −52 e, based on the 3RYF structure in the Protein Data Bank (PDB), valid at physiological pH (van den Heuvel et al., 2007). About half of this charge is located on C-termini of both tubulin monomers. MTs electrostatic properties, reviewed by Kalra et al. (2020), give rise to important phenomena such as long-distance propagation of ionic signals, signal amplification and even memristive behavior (Priel et al., 2006; Priel and Tuszynski, 2008; Sataric et al., 2009; Tuszynski et al., 2020), suggesting their role as possible signal transmission lines and memristive components in cells.

If an EZ water layer forms around MTs in a similar way to that for Nafion, this would mean that chemical reactions and interactions with other components of the cytoplasm would be influenced by it because of the effects on electrical repulsion and this could also help explain part of the environmental effects on cellular behavior since, as mentioned above, ionic concentrations influence the EZ size. This could be the case, for example, of cancer cells because the tumor microenvironment is very different from the physiological one (Mbeunkui and Johann, 2009; Hanahan and Weinberg, 2011; Hink and Nathke, 2014) with some important characteristics such as pH, transmembrane potential and ionic concentrations being different in cancer cells compared to normal cells (Webb et al., 2011). These characteristics are also involved in the determination of the EZ size (Mercado-Uribe et al., 2021). The so-called Warburg effect, which is prominently demonstrated in cancer cells, represents a switch in the major energy production from aerobic respiration to glycolysis even if in the absence of hypoxic conditions (Warburg, 1956), has been suggested to be influenced by ordered water (Pokorný et al., 2014) and experimental results suggest that ordered water layers extension around mitochondria can be modulated by light and has implications in ATP synthesis (Passerella et al., 1984; Sommer et al., 2008; Sommer et al., 2011; Sommer et al., 2015). Moreover, long-range static electric fields on the order of microns were measured from mitochondrial surfaces (Tyner et al., 2007), suggesting that membranes or other highly charged structures can generate long-range interactions which could be involved in biomolecular recognition (Preto et al., 2015). In addition, other cytoplasmic ions, e.g., K^+^ and Mg^2+^, influence MT polymerization (Olmsted and Borisy, 1973; Borisy et al., 1975; Lee and Timasheff, 1977) and since they also influence the EZ’s size, their influence on repulsion forces could be linked to the reaction rates. In addition, it has been shown that tubes made from hydrophilic materials, when immersed in water, generate a flow through themselves (Pollack, 2013). Hence, since some biomolecules such as proteins can access the MT lumen (Garvalov et al., 2006; Coombes et al., 2016) their motion inside it could be influenced by the EZ water. Finally, anesthetics, which act by binding with MTs, were able to modify the EZ size in experiments done with Nafion (Kundacina et al., 2016) so if something similar happens also with MTs this could imply a fundamental role of water in the emerging of consciousness and could support the shielding role proposed by Hameroff and Penrose in the context of the Orch OR theory (Hameroff and Penrose, 2003; Hameroff and Penrose, 2014).

The aim of this paper is therefore to provide an estimation for the EZ water formed around microtubules (MTs) in order to study the repulsion of tubulin dimers from MTs, using an approach based on the linearized Poisson-Boltzmann (LPB) equation. Moreover, since tubulin can assemble into different structures including flat sheets and macrotubes (Unger et al., 1990), the tubulin-tubulin and the tubulin-sheet interactions are also modelled in this paper.

## Materials and Methods

A good model for the evolution of the EZ was recently proposed by Mercado-Uribe et al. (2021). The approach is based on a 1-D Langevin equation describing the motion of a microparticle caused by a charged interface:

$$m\overset{..}{x}\left(t\right)=F\left(x\right)-\xi \dot{x}\left(t\right)+f\left(t\right)$$ (1)

where m and x(t) represent the particle’s mass and position, respectively. F(x) represents the electric force felt by the molecule at a distance x to the surface, ξ = 6πηa is the friction coefficient according to the Stokes approximation where η is the medium’s viscosity and a is the particle radius and f(t) is a stochastic force of zero mean value.

To fit the EZ evolution to the time-dependent data, the force was assumed to be in the following form (Mercado-Uribe et al., 2021):

$$\frac{F\left(x\right)}{\xi }\mathrm{=K}e^{\mathrm{-\kappa x}}$$ (2)

Authors have not dealt with the origin of this force, although they stated that they support the electrophoretic origin of the EZ and F(x) it is related to an electric field experienced by the molecule. Values for K and κ were obtained by fitting the data according to the analytical solution of Eq. 1 (Mercado-Uribe et al., 2021).

The force is very similar to the one obtained from the Poisson-Boltzmann (PB) equation because of the following properties: 1) it leads to an exponential decay over time (Mercado-Uribe et al., 2021), and 2) it generates ionic screening effects (Zheng and Pollack, 2008; Mercado-Uribe et al., 2021). Moreover, the force field within the EZ was experimentally measured, revealing a decreasing value of the force as a function of distance (Chen et al., 2012). The main difference is the length scale involved: the PB effect is expected to die down over a few microns at most while the EZ extent can reach hundreds of microns or even millimeters. Since the ordered water layers extension in cell seem to be in the nanometer scale (Sommer et al., 2008) the PB effect could be a starting point to model the EZ phenomenon in biological environments and it was also used for the Nafion case (Esplandiu et al., 2020). From these considerations, we take the linearized PB (LPB) equation used by Eakins et al. (2021) for the voltage V around a MT:

$$\nabla \cdot \left(\varepsilon_{r}\mathrm{\nabla V}\right)=\frac{2eN_{A}c_{s}}{\varepsilon_{0}}\frac{\mathrm{eV}}{k_{B}T}$$ (3)

in which ε_0_ and ε_r_ are the vacuum and relative permittivity, respectively, e is the elementary charge, N_A_ is the Avogadro number, c_S_ is the solution’s ionic concentration, k_B_ is the Boltzmann constant and T is the absolute temperature. The linearization can be used when the potential is below the thermal voltage (V_T_ ≈ −25 mV) and for MTs is a good approximation with respect to numerical solutions of the non-linear PB equation (Eakins et al., 2021). Since Eq. 3 has an analytical solution for particular geometries, the MT-tubulin, tubulin-tubulin and tubulin-sheet interactions are studied for two vastly different KCl concentrations (10 and 160 mM) in water (*η* = 10–3 Pa*s, ε_r_ = 78.3) at T = 20°C, according to the following workflow:

• Find an analytical solution of Eq. 3 to obtain the electric field E(x) around the repelling surface;
• Obtain an estimate of the force F(x) acting on the tubulin dimer according to the following Equation:

$$F\left(x\right)=Q_{\mathrm{eff}}E\left(x\right)$$ (4)

where Q_eff_ is the effective charge of the tubulin dimer at a given ionic concentration, estimated according to findings of Eakins et al. (2021);

• Find a numerical solution of the Equation:

$$m\overset{..}{x}\left(t\right)\mathrm{=F}\left(x\right)-\xi \dot{x}\left(t\right)$$ (5)

and an estimation of the EZ. Eq. 5 describes the motion of a single tubulin probe repelled from a surface because it is subjected to the repelling force F(x) and the friction force ξ $\dot{x}\left(t\right)$ and provides the estimation of the EZ evolution over time, in a similar way as done by Mercado-Uribe et al. (2021).

• Generate an estimate of the involved energy as a function of the distance by multiplying the voltage with the tubulin effective charge and calculating the difference with respect to the starting position.

## Results

### Microtubule–Tubulin Interaction

Considering the MT as an infinite cylinder and using cylindrical coordinates to express the voltage around the MT, starting from Eq. 3 we have (Eakins et al., 2021):

$$\frac{1}{r}\frac{d}{\mathrm{dr}}\left(r\varepsilon_{r}\frac{\mathrm{dV}}{\mathrm{dr}}\right)=\frac{2eN_{A}c_{s}}{\varepsilon_{0}}\frac{\mathrm{eV}}{k_{B}T}$$ (6)

Applying the same boundary conditions as the one used by Eakins et al. (2021), leads to the following solution for the voltage around the MT:

$$V\left(r\right)=\frac{\sigma \lambda_{D}}{\varepsilon_{0}\varepsilon_{r}}\frac{R}{\mathrm{R+d}}\frac{K_{0}\left(\mathrm{r/}\lambda_{D}\right)}{K_{1}\left(\left(\mathrm{R+d}\right)/\lambda_{D}\right)}$$ (7)

Which subsequently leads to the electric field distribution as a function of radial distance:

$$E\left(r\right)=\frac{\sigma }{\varepsilon_{0}\varepsilon_{r}}\frac{R}{\mathrm{R+d}}\frac{K_{1}\left(\mathrm{r/}\lambda_{D}\right)}{K_{1}\left(\left(\mathrm{R+d}\right)/\lambda_{D}\right)}$$ (8)

In Eqs 7, 8 R is the MT’s external radius (12.5 nm), d is the thickness of the Stern layer [0.33 nm, equal to the K^+^ ion radius, used as correction factor for ionic size (Eakins et al., 2021)], K_0_ and K_1_ are the modified Bessel functions of the second kind of zeroth and first order, respectively, σ is the surface charge density and λ_D_ the Debye length, calculated as:

$$\lambda_{D}=\frac{1}{\kappa }=\sqrt{\frac{\varepsilon_{0}\varepsilon_{r}k_{B}T}{2N_{A}c_{s}e^{2}}}$$ (9)

To use cylindrical geometry and take into account the charge of the external surface and the charge of the C-termini, which protrude from the MT, the estimation for the surface charge can be made as follows:

$$\mathrm{\sigma =}\sigma_{\mathrm{out}}\frac{A_{\mathrm{out}}}{A_{\mathrm{tot}}}+\sigma_{\mathrm{CT}}\frac{2A_{\mathrm{CT}}}{A_{\mathrm{tot}}}=-0.102\frac{C}{m^{2}}$$ (10)

In Eq. 10 an estimate is made recalling that A = Q/σ and assuming a charge of -25e for the outer surface and -11e for each C-terminus (Eakins et al., 2021). The tubulin dimer is modelled as a sphere of radius a = 2.71 nm equivalent to an ellipsoid of semi axis 2 × 2.5 × 4 nm. Eq. 5 is numerically solved assuming as initial conditions r (0) = R + d or r (0) = R+a and $\dot{r}$ (0) = 0. The position r (0) = R + d refers to the situation in which a tubulin dimer detaches from the MT because it represents the first point for which our potential is defined. The position r (0) = R+a refers to a tubulin dimer attached (not bound) to the MT external wall: since the dimer is modelled as a sphere its center of mass will be distant a from the MT wall. Both conditions are studied for concentrations of 10 and 160 mM to reproduce a lower concentration similar to *in vitro* studies and an higher one closer to cytoplasmic conditions ([K^+^] is around 140 mM in cells). Having estimated the EZ, the electrostatic energy as a function of distance is:

$$U\left(r\right)=Q_{\mathrm{eff}}\frac{\sigma \lambda_{D}}{\varepsilon_{0}\varepsilon_{r}}\frac{R}{\mathrm{R+d}}\frac{K_{0}\left(\mathrm{r/}\lambda_{D}\right)}{K_{1}\left(\left(\mathrm{R+d}\right)/\lambda_{D}\right)}$$ (11)

**FIGURE 1:**
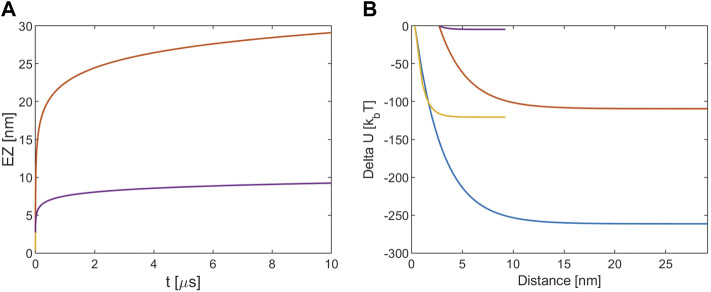
**(A)** EZ from MT. **(B)** Electrostatic potential energy variation. The blue and red lines refer to [KCl] = 10 mM, r (0) = R + d and r (0) = R+a, respectively. The yellow and violet lines refer to [KCl] = 160 mM, r (0) = R + d and r (0) = R+a, respectively. Note that for the EZ the blue and the yellow lines are masked by the red and violet one because processes times are almost identical.

Where Q_eff_ is the effective charge of the tubulin dimer in the solution. To estimate it we take the tubulin bare charge at pH = 7, equal to −52 e, and we add to it the positive charge from counterions bound to the MT complex calculated by Eakins et al. (2021). In this way we obtain Q_eff_ = −17e for 10 mM concentration and −29 e for 160 mM one. The energy variation at a distance r is calculated by subtracting the value of the energy at the starting point from the energy value at r, both calculated with Eq. 11. Results are shown in Figure 1. All plots show the relative distance between the tubulin dimer and the MT instead of the absolute r coordinate.

From Figure 1A it is possible to note that with 10 mM ionic concentration the EZ size is about 30 nm while for 160 mM the EZ is lower and the timescale for all the processes is of the order of μs. The lower EZ of the two latter cases is due to the fact that at higher ions concentrations the screening is stronger and the EZ is reduced. Figure 1B shows that the energy variation is much lower if the considered starting point is the particle radius: that is due to the fact that the Debye length at this concentration is about 3 nm, so the energy decay in the first nm away from the MT surface is important. The ionic screening reduces also the energy variation and since for the 160 mM case the Debye length is about 7.5 Å the energy estimated is very low if r (0) is assumed equal to R+a. In general the energy falls in the k_B_T-hundreds of k_B_T ranges, so it is comparable to the estimations of the free energy change involved in transferring a dimer from the MT lattice to the surrounding medium (VanBuren et al., 2002; Sept et al., 2003; VanBuren et al., 2005).

### Tubulin–Tubulin Interaction

To study the interaction between two tubulin dimers is convenient to model them again as spheres of radii 2.71 nm in order to apply a spherical symmetry approximation. The LPB equation expressing the voltage around the tubulin dimer in spherical coordinates becomes:

$$\frac{1}{r^{2}}\frac{d}{\mathrm{dr}}\left(\varepsilon_{r}r^{2}\frac{\mathrm{dV}}{\mathrm{dr}}\right)=\frac{2e^{2}N_{A}c_{s}}{\varepsilon_{0}k_{B}T}V$$ (12)

By applying Gauss’ law at the Stern layer (r = R + d) we find:

$$-\frac{\mathrm{dV}}{\mathrm{dr}}=\frac{\sigma }{\varepsilon_{0}\varepsilon_{r}}\frac{R^{2}}{{\left(\mathrm{R+d}\right)}^{2}}$$ (13)

As a second boundary condition we suppose the electric field to be null at infinity. These two conditions lead to the following analytical solution:

$$V\left(r\right)=\frac{\sigma }{\varepsilon_{0}\varepsilon_{r}}\frac{R^{2}}{{\left(\mathrm{R+d}\right)}^{2}}r^{\mathrm{-1/2}}\frac{K_{\mathrm{1/2}}\left(\mathrm{r/}\lambda_{D}\right)}{g\left(\mathrm{R+d}\right)}$$ (14)

which leads to the electric field’s radial distribution given by:

$$E\left(r\right)=\frac{\sigma }{\varepsilon_{0}\varepsilon_{r}}\frac{R^{2}}{{\left(\mathrm{R+d}\right)}^{2}}\frac{g\left(r\right)}{g\left(\mathrm{R+d}\right)}$$ (15)

where the function g(z) is defined as:

$$g\left(z\right)=\left\{\frac{z^{\mathrm{-3/2}}}{2}K_{\mathrm{1/2}}\left(\mathrm{z/}\lambda_{D}\right)+\frac{r^{\mathrm{-1/2}}}{\lambda_{D}}\left[K_{\mathrm{3/2}}\left(\mathrm{z/}\lambda_{D}\right)-\frac{\lambda_{D}}{2z}K_{\mathrm{1/2}}\left(\mathrm{z/}\lambda_{D}\right)\right]\right\}$$ (16)

In Eqs 13–16 R is the tubulin radius, K_i_ are the modified Bessel functions of the *i*th order and the surface charge is calculated as:

σ=Qbare4πR2=-0.090 Cm2 (17)

Where the bare charge of the tubulin dimer is assumed as Q_bare_ = −52 e. Now the potential energy is:

$$U\left(r\right)=Q_{\mathrm{eff}}\frac{\sigma }{\varepsilon_{0}\varepsilon_{r}}\frac{R^{2}}{{\left(\mathrm{R+d}\right)}^{2}}r^{\mathrm{-1/2}}\frac{K_{\mathrm{1/2}}\left(\mathrm{r/}\lambda_{D}\right)}{g\left(\mathrm{R+d}\right)}$$ (18)

**FIGURE 2:**
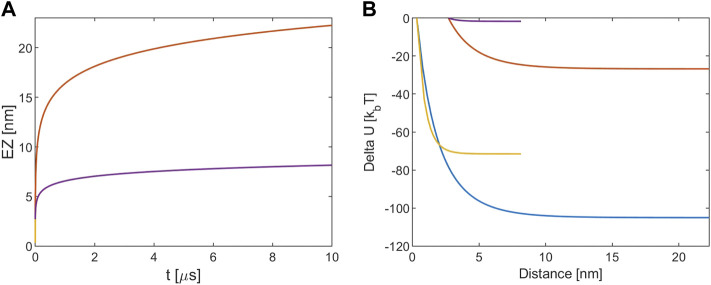
**(A)** EZ between tubulin dimers. **(B)** Electrostatic potential energy variation. The blue and red lines refer to [KCl] = 10 mM, r (0) = R + d and r (0) = R+a, respectively. The yellow and violet lines refer to [KCl] = 160 mM, r (0) = R + d and r (0) = R+a, respectively. Note that for the EZ the blue and the yellow lines are masked by the red and violet one because processes times are almost identical.

Here, both Q_eff_ and the energy variation are estimated as before. Simulations are performed with similar initial conditions as before, the only difference is that R now is the tubulin radius instead of the MT one and the results of our simulations are shown in Figure 2. Again, all plots show the relative distance between the dimers instead of the absolute r coordinate.

Figure 2A shows that the EZ size for the 10 mM case extends for about 20 nm over 10 μs, so with respect to the MT-tubulin interaction at the same ionic concentration the repulsion is lower. Again, at 160 mM the electrostatic repulsion leads to a lower EZ with respect to the 10 mM case and to the one estimated for the corresponding MT-tubulin interaction. Figure 2B confirms the previous results because as before the difference in the Debye length of the solutions is reflected in the energy variations, such that the assumption of r (0) = R+a leads to a very low energy release with respect to the other values.

### Tubulin Sheet–Tubulin Interaction

In this case the LPB equation is used to express the voltage generated by the planar tubulin sheet in Cartesian coordinates. Considering the sheet as an infinite plane allows to have a 1-D equation for the potential:

$$\varepsilon_{r}\frac{d^{2}V}{dx^{2}}=\frac{2eN_{A}c_{s}}{\varepsilon_{0}}\frac{\mathrm{eV}}{k_{B}T}$$ (19)

By applying Gauss’ law at x = d and assuming the electric field to be null for x going to infinity as boundary conditions, the analytical solution of Eq. 19 is found as:

$$V\left(x\right)=\frac{\sigma \lambda_{D}}{2\varepsilon_{0}\varepsilon_{r}}e^{-\left(\mathrm{x-d}\right)/\lambda_{D}}$$ (20)

which leads to the electric field distribution as a function of distance given by:

$$E\left(x\right)=\frac{\sigma }{2\varepsilon_{0}\varepsilon_{r}}e^{-\left(\mathrm{x-d}\right)/\lambda_{D}}$$ (21)

In this case the surface charge density is σ = −0.090 C/m^2^, calculated in the same way as in the tubulin-tubulin interaction. Finally, the electrostatic energy becomes:

$$U\left(x\right)=Q_{\mathrm{eff}}\frac{\sigma \lambda_{D}}{2\varepsilon_{0}\varepsilon_{r}}e^{-\left(\mathrm{x-d}\right)/\lambda_{D}}$$ (22)

**FIGURE 3:**
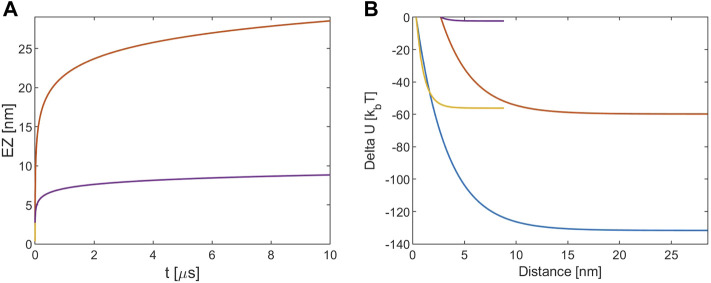
**(A)** EZ from tubulin sheet. **(B)** Electrostatic potential energy variation. The blue and red lines refer to [KCl] = 10 mM, r (0) = R + d and r (0) = R+a, respectively. The yellow and violet lines refer to [KCl] = 160 mM, r (0) = R + d and r (0) = R+a, respectively. Note that for the EZ the blue and the yellow lines are masked by the red and violet one because processes times are almost identical.

Where the effective charge of the tubulin dimer is estimated as before. The energy variation calculation procedure is the same and the applied initial conditions are x (0) = a or x (0) = d and null initial velocity. Results are shown in Figure 3.

Figure 3A shows that the EZ size is close to the one obtained in the MT-tubulin interaction for all the cases and the process has the same timescale. On the other hand, this time the energies involved are about one half of those in that case, as depicted in Figure 3B The Debye length influences therefore all the processes in a similar way.

## Discussion

Based on the results of the simulations reported in this paper, it is possible to state the following predictions regarding the effects of EZ water on microtubules in ionic solutions:

• The ionic screening effect means that at a lower KCl concentration the counterion screening is reduced so the voltage is higher. This leads to a stronger force felt by the particle and thus to a larger EZ, which is consistent with experimental results reported for Nafion (Zheng and Pollack, 2008; Mercado-Uribe et al., 2021).
• The evolution of EZ around MTs is very fast at the beginning of the process (a couple of μs) and then it slows down significantly, meaning that the process has a timescale on the order of tens of microseconds.
• The energy released by the system varies from several k_B_T to hundreds of k_B_T depending on the simulation conditions. In particular most of the energy is released in the first 3 nm and this is due to the fact that the Debye length, which is the decay constant associated to the PB potential, is of the order of 1 nm.

The simulations reported here were made using the LPB equation, which was found to be a good approximation for higher concentrations but led to an overestimation of the voltage for lower ones with respect to numerical simulations conducted using a modified non-linear PB equation to take into account the ionic size (Eakins et al., 2021).

A possible explanation of the EZ extension, i.e., that EZ with Nafion is greater than expected from the PB equation, could be related to the fact that EZ water also excludes protons, leading to the formation of a detectable Proton Zone (PZ) afterwards (Wang and Pollack, 2021) that is also predicted by quantum electrodynamics considerations (Del Giudice et al., 2015). This could also happen to all the other positive ions in the solution, leading to a zone in which ion concentrations are very low. By looking at Eq. 9 is possible to note that this fact would imply a higher “local” Debye length close to the repelling surface and as a consequence a reduced screening effect and a longer range of interaction than expected with respect to bulk water. Probably this exclusion of the positive ions is not as efficient as the exclusion of negative particles and so some positive ions penetrate into the EZ, which would explain why the addition of positive ions reduces the EZ and why the potential in the EZ is more screened when positive ions are added to the solution (Zheng and Pollack, 2008). In addition to this, since the EZ has a higher refractive index than water (Pollack, 2013), it also has an higher relative dielectric constant and thus a larger Debye length (although this effect on the Debye length is very small). These considerations would explain also how the electromagnetic radiation enhances the EZ extension. From experimental observation IR radiation increases not only the EZ but also the PZ size (Wang and Pollack, 2021): therefore, even more protons are excluded and the “local” Debye length would increase even more. Future works will be focused on estimate how many ions are excluded in order to improve the model proposed here and to use it to simulate the effect of IR radiation.

Among the experimental techniques that could be used to investigate the EZ properties Aquaphotomics could be of great interest. Aquaphotomics is a new discipline in which light with different frequencies is used to investigate the structure of water (Tsenkova et al., 2018; Muncan and Tsenkova, 2019) and thus infer the system’s intrinsic properties. This technique was already used to detect EZ water (Renati et al., 2019) and demonstrated that electromagnetic radiation changes the molecular network of water (Muncan and Tsenkova, 2019). Applying Aquaphotomics could be of interest to test hypotheses regarding the EZ formation not only in the case of Nafion but also for other compounds, including biological polymers like MTs. Our results coupled with experiments could help explaining environmental influence on MTs polymerization and dynamics. In particular the role of ions, that as said before influence the process a lot (Olmsted and Borisy, 1973; Borisy et al., 1975; Lee and Timasheff, 1977): part of their effect can be due to the charge screening showed here. A better understanding of environmental effects on MTs dynamics could help gaining more information on cells pathological conditions, leading therefore to the design of novel therapies or novel ways to target unhealthy cells. In addition electromagnetic radiation can generate collective excitations and long-range interactions in MTs according to theoretical calculations (Sukhov et al., 2013; Fahri and Dogariu, 2021) and can act on MTs polymerization (Tulub and Stefanov, 2004): these effects could be related to the modifications of the surrounding water observed by Pollack. Electromagnetic radiation seems also to influence drug absorption by the cell by its action on interfacial water layers (Sommer et al., 2010) so this results can provide a first insight on the role of light in chemotherapy. In addition, the possibility of tuning the ordered water layers extension and therefore change the drug absorption could give rise to more efficient and mini invasive therapies if devices that can selectively act on pathological cells or their components will be developed. Finally, also heavy water (D_2_O) has effects on MTs dynamics (Houston et al., 1974; Chakrabarti et al., 1999; Panda et al., 2000) so if its properties influence the water ordering repulsions could change. Future works will be therefore focused on EZ, electromagnetic radiation and environmental conditions effects on MTs polymerization and dynamics.

Finally, if such a depletion zone origins, a change in the refractive index around the MT is expected [this happens with Nafion (Pollack, 2013)]. The way in which the refractive index changes would help explaining the MT behavior as an antenna. From recent results on local pH changes around MTs (Kalra et al., 2022) we expect a gradual change of the refractive index which would imply the MT acting as a graded-index fiber for which the pH and the ionic concentration dependence are significant. Further investigations on the role of water in biological compound are therefore of great interest to understand how biological systems work because the water abundance in biological organisms makes it a fundamental element in life and its role is not yet fully understood.

## Data Availability

The original contributions presented in the study are included in the article/Supplementary Material, further inquiries can be directed to the corresponding author.
